# Does Proteomic Mirror Reflect Clinical Characteristics of Obesity?

**DOI:** 10.3390/jpm11020064

**Published:** 2021-01-21

**Authors:** Olga I. Kiseleva, Viktoriia A. Arzumanian, Ekaterina V. Poverennaya, Mikhail A. Pyatnitskiy, Ekaterina V. Ilgisonis, Victor G. Zgoda, Oksana A. Plotnikova, Khaider K. Sharafetdinov, Andrey V. Lisitsa, Victor A. Tutelyan, Dmitry B. Nikityuk, Alexander I. Archakov, Elena A. Ponomarenko

**Affiliations:** 1 Institute of Biomedical Chemistry, Pogodinskaya Street 10/8, 119121 Moscow, Russia; viktoriia.arzumanian@ibmc.msk.ru (V.A.A.); k.poverennaya@ibmc.msk.ru (E.V.P.); mpyat@ibmc.msk.ru (M.A.P.); ilgisonis@ibmc.msk.ru (E.V.I.); vic@ibmh.msk.su (V.G.Z.); fox@ibmh.msk.su (A.V.L.); alexander.archakov@ibmc.msk.ru (A.I.A.); elena.ponomarenko@ibmc.msk.ru (E.A.P.); 2 Federal Research Centre of Nutrition, Biotechnology and Food Safety, Russian Academy of Sciences, Ustinsky Passage 2/14, 109240 Moscow, Russia; plotnikova@ion.ru (O.A.P.); sharafetdinov@ion.ru (K.K.S.); tutelyan@ion.ru (V.A.T.); nikitjuk@ion.ru (D.B.N.); 3 Russian Medical Academy of Continuing Professional Education, Ministry of Health of the Russian Federation, Barrikadnaya Street 2/1, 125993 Moscow, Russia; 4 I.M. Sechenov First Moscow State Medical University (Sechenov University), Ministry of Health of the Russian Federation, Trubetskaya Street 8/2, 119991 Moscow, Russia

**Keywords:** obesity, BMI, blood tests, proteomics, mass spectrometry

## Abstract

Obesity is a frightening chronic disease, which has tripled since 1975. It is not expected to slow down staying one of the leading cases of preventable death and resulting in an increased clinical and economic burden. Poor lifestyle choices and excessive intake of “cheap calories” are major contributors to obesity, triggering type 2 diabetes, cardiovascular diseases, and other comorbidities. Understanding the molecular mechanisms responsible for development of obesity is essential as it might result in the introducing of anti-obesity targets and early-stage obesity biomarkers, allowing the distinction between metabolic syndromes. The complex nature of this disease, coupled with the phenomenon of metabolically healthy obesity, inspired us to perform data-centric, hypothesis-generating pilot research, aimed to find correlations between parameters of classic clinical blood tests and proteomic profiles of 104 lean and obese subjects. As the result, we assembled patterns of proteins, which presence or absence allows predicting the weight of the patient fairly well. We believe that such proteomic patterns with high prediction power should facilitate the translation of potential candidates into biomarkers of clinical use for early-stage stratification of obesity therapy.

## 1. Introduction

Obesity in most cases is blatantly visible by the unaided eye. Paradoxically, at the same time both clinicians and citizens tend to ignore this pathology, acquiring the scale of “globesity” [1,2]. Being a generally preventable disease, obesity, resulting from the excess of body fat, often entails the development of 50+ various pathologies, significant disability, and premature death [3].

The pathogenesis of obesity involves the interaction of genetic, environmental, and behavioral factors [4]. Each time, figuring out the characteristic features in the biomedical portrait of obesity, scientists are trying to resolve the nature vs nurture debate [5]. The multifactorial nature and high comorbidity of obesity make it difficult to understand the clear molecular nature of this disease. Moreover, about a third of obese patients are “metabolically healthy” with little or no evidence of metabolic syndrome. There are four central features (insulin resistance, increased visceral fat, atherogenic dyslipidemia, and endothelial dysfunction), which make up the essential definition for metabolic syndrome. Among them, only the first two are obligatory [6].

However, metabolic syndrome is not the one and only way to identify individuals with increased risk of cardiovascular diseases and diabetes, as well as other comorbidities. Reliable identification of individuals with a significant risk of endocrine or cardiovascular complications requires assessment methods taking into account orthogonal factors (e.g., family history, age, sex, smoking, and other crucial parameters) [6].

Importantly, the “healthy” phenotype of an obese individual with no metabolic aberrations is not constant. Thus, the metabolism of half of such patients ceases being “healthy” in ca. 10 years [7]. It means that early diagnostics and intervening even for “metabolically healthy obesity” is crucial. The dynamic nature of obesity makes it even more difficult to find out meaningful differences between normal state, metabolically healthy, and unhealthy obesity.

Despite the apparent obviousness of strategies for treatment and prevention of obesity, unfortunately, in the long term, they demonstrate low efficiency, primarily because standard pharmacological solutions and significant behavioral changes regarding nutrition and activity, in most cases, do not take root in the modus vivendi of the patients.

A major diagnostic criterion of obesity is body mass index (BMI), expressed by a person’s weight divided by the square of his or her height. BMI reliably indicates the anthropometric condition for the overwhelming majority of cases; however, it does not accurately reflect the severity of the health risks [8,9]. The same could be stated for traditional laboratory tests, including monitoring of triglycerides and lipid profiles [10].

The dynamic and multifactorial nature of obesity may be the reason why there are still no biomarkers approved by the FDA or other reputable organizations that could be effectively used to diagnose this disease in personalized—not “one size fits all”—mode.

High-throughput technologies accelerated life science dramatically: the speed of reading the sequences of biological macromolecules is no longer a bottleneck for unraveling the mechanisms of health and disease [11]. Since the sequencing of DNA emerged, a wide range of projects aimed at establishing genetic markers of various medical conditions were performed, and obesity is no exception. Several large-scale genomic studies (e.g., DiOGenes, which explored biological samples from 350 European families [12,13]) paved the way for a further selection of nutritional recommendations through understanding the dynamics of weight maintenance based on the uniqueness of a particular patient [14].

Technical progress in the exploration of the proteome, the final level of transmission of biological information and predecessor of the metabolome, achieved during the last decade has provided a base for illuminating risks and improving current therapeutic strategies [15]. Proteomics allows the development of a personalized molecular profile that takes into account the pattern of biomarkers. This “molecular mirror” reflects all significant processes in the body, including systemic chronic inflammation, associated with obesity [16,17,18].

In the study, we used a proteomics approach to get a panoramic picture of the proteome of patients with respect to their weight status. To the best of our knowledge, we provide the first evidence that qualitative plasma protein landscape significantly differs from classic clinical parameters on an issue of obesity. We highlighted proteins, which could play a significant role in the development of obesity and obesity-associated disease and should be additionally explored as a prominent tool to improve risk stratification.

## 2. Materials and Methods

### 2.1. Sample Collection

One hundred and four human plasma samples were obtained from the patients of the Clinic of “Federal Research Centre of Nutrition, Biotechnology and Food Safety” (Moscow, Russia).

All study participants gave informed consent confirming their willingness to participate in the research. All procedures performed in studies involving human participants were under the ethical standards of the institutional or national research committee and with the 1964 Helsinki declaration and its later amendments or comparable ethical standards. The study was approved by the relevant ethical review committee of the Federal Research Centre of Nutrition, Biotechnology and Food Safety (protocol #4 from 15 June 2018).

The present study included 104 individuals in accordance with the inclusion and exclusion criteria. The inclusion criteria were the age of the study participants from 18 to 45 years, BMI from 18.5, absence of diagnosed somatic and mental disorders.

Individuals younger than 18 and older than 45 years were excluded from the study, as well as pregnant/breastfeeding patients, patients with mental disorders, identified cancer, cardiovascular and any gastrointestinal diseases, other somatic disorders, and recent (6 months) weight loss.

The patients were divided into groups according to their body mass indexes. BMI, calculated as the mass of the individual in kilograms divided by his/her height in meters squared, is one of the most popular metrics to characterize body condition [19].

Five groups of patients (Table 1) were enrolled for this study: controls (NORM, BMI = 18.5–24.9), overweight patients (OW, BMI = 25.0–29.9), and patients with obesity stage 1 (OB1, BMI = 30.0–34.9), 2 (OB2, BMI = 35.0–39.9), and 3 (OB3, BMI > 40.0).

Venous blood samples were collected into EDTA tubes after overnight fasting and centrifuged at 1500× *g*, for 10 min at room temperature. Plasma fractions were stored at −80 °C in cryotubes until processing. Samples were randomized prior to proteomic investigation to avoid potential batch effects.

### 2.2. Anthropometric and Clinical Tests

#### 2.2.1. Anthropometric Tests

The BMIs of the patients were evaluated according to the standard formula [19]. The weight distributions were measured using the bioelectrical impedance analysis method.

#### 2.2.2. Biochemical Blood Test and Complete Blood Count

Serum levels of fasting plasma glucose, triglycerides, high-density lipoprotein, low-density lipoprotein, cholesterol, alanine aminotransferase, aspartate aminotransferase, *γ*-glutamyl transpeptidase, alkaline phosphatase, uric acid, urea, creatinine, albumin, bilirubin, etc. were determined according to standard protocols. Blood levels of hemoglobin, hematocrit, and blood cell indexes were established according to standard protocols [20].

Results of anthropometric and blood tests are provided in Supplementary Table S1.

### 2.3. Sample Preparation

#### 2.3.1. The Depletion of Blood Plasma

The immunoaffinity depletion of the high abundance plasma proteins (albumin and IgG) was used to enhance the detection of lower abundance but more insightful proteins in further shotgun proteomic analysis. For plasma depletion, we used ProteoPrep Kit (Sigma-Aldrich, St. Louis, MO, USA). The depletion was carried out following the manufacturer’s instructions [21].

#### 2.3.2. Trypsinolysis of Depleted Plasma

The depleted blood plasma samples (175 μg of total protein) were in-solution digested in accordance with a standard protocol [22]. In brief, proteins were denatured and reduced with a solution containing sodium deoxycholate, tris-2-carboxyethyl-phosphine hydrochloride, and 1,4-dithiothreitol, and further alkylated with vinylpyridine. Trypsin was added to the sample (trypsin/total protein = 1/100) and then incubated within 2 h at a temperature of 44 °C. After 2 h, an aliquot of trypsin was added and then incubated for 2 h at 37 °C. Trypsinolysis was quenched by adding formic acid to each sample to a final concentration of 5%, then a mixture of peptides was centrifuged at 10,000 rpm within 15 min. The supernatant was collected and subjected to further chromatography-mass spectrometric analysis.

### 2.4. HPLC-MS/MS Analysis

Separation and identification of the peptides were performed on an Ultimate 3000 nano-flow HPLC (Thermo Fisher Scientific, Cleveland, OH, USA) connected to Orbitrap Exactive (Thermo Fisher Scientific, Cleveland, OH, USA) mass spectrometer equipped with a Nanospray Flex NG ion source (Thermo Fisher Scientific, Cleveland, OH, USA). Peptide separation was carried out on an RP-HPLC column Zorbax 300SB-C18 (C18 particle size of 3.5 μm, inner diameter of 75 μm and length of 150 mm, Acclaim^®^ PepMap™ RSLC, Thermo Fisher Scientific, Cleveland, OH, USA) using a linear 90-min gradient from 95% solvent A (0.1% formic acid) and 5% solvent B (0.1% formic acid, 80% acetonitrile) to 60% solvent B over 95 min at a flow rate of 0.3 μL/min.

Mass spectra were registered in the positive ion mode. Data was acquired in the Orbitrap Exactive analyzer with a resolution of 70,000 (at *m*/*z* 400) for MS and 15,000 (*m*/*z* 400) for MS/MS scans. For peptide fragmentation higher energy collisional dissociation (HCD) was used, the signal threshold was set to 17,500 for an isolation window of 1 *m*/*z* and the first mass of HCD spectra was set to 100 *m*/*z*. The collision energy was set to 35%. Fragmented precursors were dynamically excluded from targeting for 10 s. Singly charged ions and ions with not defined charge states were excluded from triggering MS/MS scans. Three LC-MS/MS repetitions were performed for each plasma sample.

### 2.5. Interpretation of Experimental Data

Raw files were converted into .mgf files by MSConvert (v. 3.0). Each of the 312 mgf files containing the feature list for protein identification was processed by SearchGUI software (v. 4.0.4 [23]) using three search engines (X!Tandem, MS-GF+, OMMSA) against SwissProt library of human canonical and alternatively spliced protein sequences in automatic mode [24]. Trypsin was specified as the proteolytic enzyme; maximum of 2 missing cleavages were allowed. Pyridylethylation (C) was used as a constant modification, and oxidation of methionine was set as a variable one. Charge states of +2, +3, and +4 were selected as parent ions. Mass tolerance was set to ±15 ppm for precursor ions and ±0.01 Da for fragment ions. The cut-off of false discovery rates for peptide-spectra matches, peptides, and proteins was ≤1%. Results were visualized in PeptideShaker (v. 2.0.5 [25]). The MS data were deposited to the ProteomeXchange Consortium via the PRIDE partner repository [26] with the dataset identifier PXD023526.

### 2.6. Statistical Analysis

Clustering analysis of clinical and anthropometric tests was performed using Ward’s method and Euclidean distance for normalized data. Clustering patterns of protein presence/absence were done using Ward’s method and Jaccard distance metric. All statistical analyses and graphics were performed using R version 4.0 [27].

Each protein of interest was annotated with its GO-terms from UniProt using ViSEAGO package [28]. We used the “2020-03” GO release and “2020_01” UniProt release.

When comparing the results of proteomic and clinical analysis, we explored publicly available data on the relationship between proteins and parameters of clinical analysis. The automatic analysis of the texts of scientific publications was performed by Scanbious platform [29,30], which visualizes semantic networks between objects of various types (names of proteins, pathological processes, etc.).

We predicted the BMI of the patient based on the pattern of presence/absence of certain proteins in his/her blood plasma using the Least Absolute Shrinkage and Selection Operator (LASSO) regression implemented in glmnet package [31]. We performed 10 iterations, each time randomly selecting 90% of the samples. For each iteration we needed to select the optimum value of LASSO tuning parameter lambda, which penalizes the sum of the absolute values of the coefficient. Optimum value of lambda was also selected by performing cross-validation (10 runs of 10-fold cross-validation cycle). The lambda with the minimum average error was selected as a lambda for the current iteration. Final model included only proteins, which were selected at every iteration (10 out of 10 times). Model performance was estimated as the median absolute error which was defined as the median of absolute differences between the true BMI and the predicted BMI.

## 3. Results and Discussion

### 3.1. Clinical Component

Much attention has been riveted on the phenomenon of metabolically healthy obesity (MHO), characterized by the absence of the metabolic abnormalities that traditionally accompany excess adiposity [32]. Thus, a substantial proportion of the obese subjects does not seem to be at an (at least temporarily, [33]) increased risk of mortality and metabolic complications of obesity. MHO is characterized by the absence of dyslipidemia, hypertension, insulin resistance, and chronic inflammation.

Moreover, lean subjects may possess abnormal metabolic parameters (exhibiting metabolically unhealthy non-obesity, MUNO) [34]. A gradient of metabolically healthy and unhealthy obese and lean phenotypes makes the revealing of abnormalities as well as relevant prevention of risks more difficult even for non-obese individuals.

**Figure 1:**
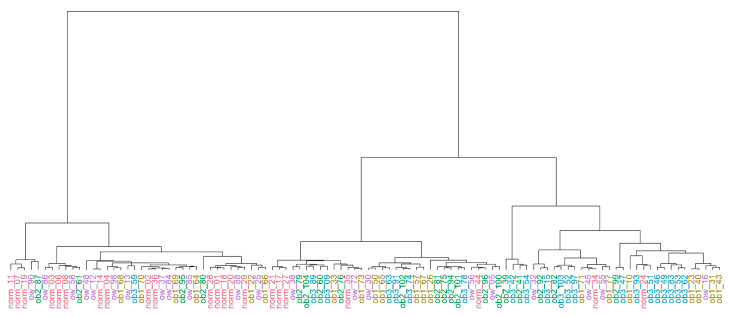
Visualization of the results of hierarchical cluster analysis of significantly different parameters of clinical and anthropometric tests performed for patients with different weight conditions. Sample IDs are presented as follows: group_number of the sample (NORM—normal weight, OW—overweight, OB1, 2, 3—obesity stage 1, 2, and 3, correspondingly).

To elucidate whether there is a bias to any of the selected extremes (four combinations of metabolic status and BMI) in our sample collection, we selected the monitored parameters of blood and anthropometric tests (Supplementary Table S1), which significantly differed between groups under study (NORM, OW, OB1, OB2, OB3). For these differed parameters, we performed a principal components analysis (Supplementary File S1) and hierarchical cluster analysis (Figure 1) using Ward’s minimum variance. The results of cluster analysis were evaluated with the Adjusted Rand Index (ARI), which reflects an agreement between two partitions: one given by the clustering process and the other defined by external criteria.

In our case, ARI was equal to 0.051, which indicates a low similarity between resulting and expected clustering. According to the obtained result, it is not possible to explicitly define the boundaries between groups of subjects with different weight conditions under study.

The impossibility to unambiguously divide patients according to their weight conditions based only on the results of clinical tests once again emphasizes the controversial and considerably challenging nature of obesity and indicates the need for orthogonal data.

In our opinion, the most promising for solving this problem will be the transition to the proteome level and multiplex assessment of the patient’s proteome landscape.

### 3.2. Proteomic Component

In total, 154 proteins were reliably identified in the entire collection of plasma samples. These proteins are predominantly associated with peptidase activity, receptor binding, and lipid transporter activity. Most of the proteins are localized in blood microparticles or plasma lipoprotein particles and vesicles, and therefore we expect stable detection of them under various mass spectrometric protocols. Of those, 36 proteins were consistently found in all plasma samples under study. A total of 138 proteins were identified in the NORM group of lean subjects. A total of 148 proteins were identified in the integrated group of overweight (OW) and obese (OB1, OB2, OB3) samples.

Next, we performed a principal components analysis (Supplementary File S1) and studied possible relationships between the pattern of presence/absence of proteins in blood plasma and the patient’s BMI using cluster analysis.

Preliminarily, unrepresentative proteins (identified in a single sample in the collection) and non-specific proteins (identified in all samples) were excluded from the calculations. The data matrix consisted of 104 rows (samples) and 101 columns (proteins).

**Figure 2:**
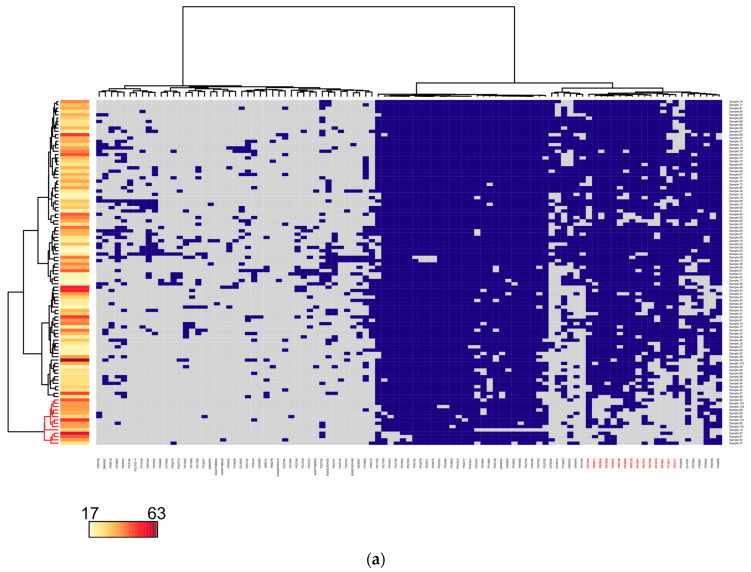

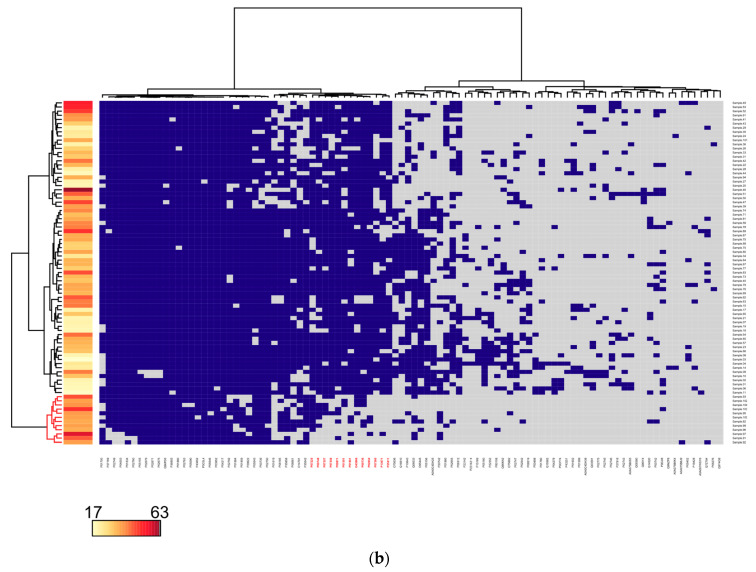
Cluster analysis of the presence/absence patterns of (**a**) 101 proteins (columns) in 104 blood plasma samples (rows) for all 104 samples under study (rows) and (**b**) 98 proteins (columns) in 83 samples, excluding plasma samples obtained from overweight individuals. The color bar indicates BMI.

The pattern of 15 proteins (namely, P07225, P00748, P07357, P07358, P09871, P01591, P01861, O43866, P00736, P02654, P13671, P25311, P01619, P01859, and P29622) allows to distinguish (Figure 2a) a group of 14 samples with increased BMI (mean 39 vs 33, *p*-value = 0.002). Moreover, 11 of them belong to the OB2 and OB3 groups, and three samples were obtained from overweight individuals. It should be noted that these three patients from OW were diagnosed with blood lipids disorder (there are seven samples with such a diagnosis in the whole OW group). Half of the samples from the OB2 and OB3 groups were also characterized by this diagnosis. As part of a pattern of 15 proteins for 5 (P07225 [35], O43866 [36,37], P02654 [38], P25311 [39,40,41], P29622 [42]) the association with the obesity was shown. It is noteworthy that five of these 15 proteins are complement components, included in two complexes: P07358, P07357, and P13671 organize membrane attack complex (MAC), that plays a key role in the innate and adaptive immune response, and P09871 and P00736 combine with serine protease to form the first component of the classical and less variable pathway of the complement system, also associated with obesity [43,44].

The collection of the samples under study contains blood plasma from overweight patients with an increased body mass index (OW), but not exceeding the threshold values required for the diagnosis of obesity, which could affect the results of cluster analysis. In this respect, we removed from consideration 21 samples from the borderline OW group, as a result, the total number of identified proteins remained practically unchanged, as well as the set of proteins common for the two—NORM and OB—groups. The updated matrix consisted of 83 rows (samples) and 98 columns (proteins) plotted with the same parameters. Clustering indices improved slightly, so for the group with high BMI its mean value was 42, and for the rest—32 (*p*-value = 0.002, Figure 2b).

The composition of the cluster with high BMI practically did not change—three samples from the OW group left, and one image from the OB2 group was added. Accordingly, the pattern of specific proteins did not change significantly, it included 13 proteins, where 12, except two immunoglobulins (P01619 and P01859) and serpin (P29622), coincide with the results of the pattern of proteins according to the all-samples clustering. New in the resulting pattern is the component of the above MAC complex—P07360.

### 3.3. BMI Prediction

**Figure 3:**
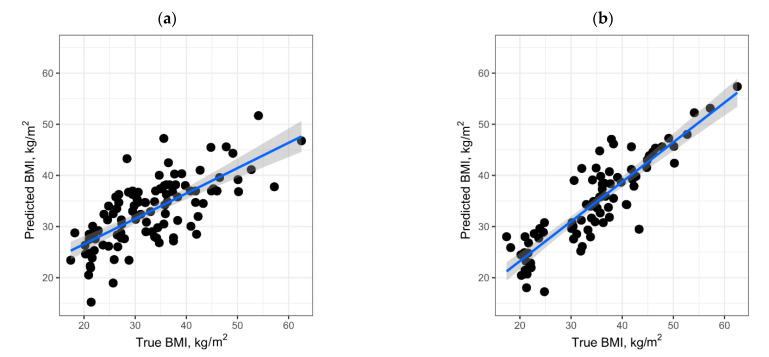
BMI prediction based on pattern of presence/absence of proteins for (**a**) all samples and for (**b**) collection without plasma samples obtained from overweight individuals.

To assess the contribution of proteins to obesity, an attempt to predict the BMI of the sample based on proteomic data was performed. For this, using the LASSO method, we built a regression model predicting the BMI of a sample according to the pattern of presence/absence of proteins in blood plasma. The model based on all data consisted of five proteins (P08185, P0DJI8, P10643, P25311, and P35858), and the median absolute error (MAE) was 5.1 kg/m^2^ (Figure 3a). At the same time, the model obtained on the basis of processing data excluding samples from the OW group showed a higher accuracy, MAE = 3.2 kg/m^2^ (Figure 3b), and the number of proteins required to build the model was 18.

It is noteworthy that the pattern of 18 proteins includes five proteins from the model with lower predictive power, as well as three previously considered proteins of the complement system (P00736, P07358, P07360) included in the MAC complex, which is indirectly associated with obesity [45].

Text-mining [29,30] performed for these proteins and their relations with pathological processes showed that 15 out of 18 proteins (Table 2) are associated to varying degrees with metabolic disorders, including obesity. For example, according to our model, the absence of sex hormone-binding globulin (P04278) correlates with increased BMI, which is confirmed by studies on its expression, where it was shown that inhibition of the corresponding gene leads to the development of obesity [46].

Summarizing the above said, we can conclude that among the reliably and reliably detected proteins [76] in blood plasma, there is a pattern that has predictive power in the issue of obesity. The minimum pattern size is five proteins. Expanding the panel increases the level of BMI prediction accuracy, which can be critical in examining borderline states in metabolically healthy obese and unhealthy lean, and also provide researchers with additional information about body composition status even when exploring protein profiles from the patients with non-obesity disorders.

We would like to stress that our intention was not to build the perfect BMI prediction model (the dataset is quite limited for this task) but rather to point to some plasma proteins likely associated with obesity when analyzed together. We suppose that the further studies needed to elaborate on this issue will also allow detection of the transition from a “metabolically healthy” phenotype of the patient with a high BMI to an “unhealthy” one.

## 4. Conclusions

According to the authors’ knowledge, no approved omics pattern has been developed to distinguish individuals at increased risk of obesity and its comorbidities. In the present study, we analyzed clinical and anthropometrical parameters of 104 subjects with different weight conditions. Each individual was also characterized by the profile of core proteins circulating through his/her blood plasma.

Our main conclusions were two-fold:

1. We demonstrated the impossibility to divide patients according to their weight conditions based only on the results of standard blood tests. Orthogonal, in our case—proteomic, data upgrades the level of understanding of the controversial nature of obesity.
2. Our overall results indicate that studies of proteins circulating in blood have the prediction power of the weight status of the patient under study. We composed two proteomic patterns (including 5 and 18 proteins, respectively), which provide additional information about the patient’s phenotype for more personalized treatment.

We strongly believe that such proteomic patterns have great potential as warning labels, signaling about obesity-associated alterations, and, thus, improving early-stage therapy of both metabolically unhealthy obese and lean individuals.

## Figures and Tables

**Table 1:** Characteristics of patients enrolled in the study.

	NORM	OW	OB1	OB2	OB3	*p*-Value ^1^
Number of patients	22 13/9 (f/m)	21 10/11 (f/m)	19 10/9 (f/m)	21 10/11 (f/m)	21 11/10 (f/m)	-
Age (years, mean ± std. deviation)	30.54 ± 5.34	32.90 ± 6.66	29.89 ± 8.16	32.62 ± 7.92	34.05 ± 6.64	0.4
Height (cm ± std. deviation)	172.65 ± 7.31	171.99 ± 11.81	170.15 ± 11.99	172.26 ± 9.74	172.08 ± 9.72	0.7
Weight (kg ± std. deviation)	64.93 ± 8.12	81.80 ± 12.24	94.44 ± 13.23	109.62 ± 13.67	140.33 ± 27.76	<0.001
BMI (kg/m^2^ ± std. deviation)	21.73 ± 1.90	27.52 ± 1.35	32.51 ± 1.69	36.81 ± 1.39	46.99 ± 5.81	<0.001

**^1^** To refute the theory that the group characteristics do not differ significantly, a *p*-value < 0.05 was used. *p*-value, calculated for age and height characteristics of groups under study, provides evidence, that differences of age and height groups are statistically insignificant. NORM—individuals with BMI = 18.5–24.9; OW—overweight patients with BMI = 25.0–29.9; OB1—patients with obesity stage 1 and BMI = 30.0–34.9; OB2—patients with obesity stage 2 and BMI = 35.0–39.9; OB3—patients with obesity stage 3 and BMI > 40.0.

**Table 2:** Proteins included into predicting pattern and their association with obesity.

#	UniProt ID	Gene Name	References	Comment on the Association with Obesity
1	A0A0C4DH25	*IGKV3D-20*	-	-
2	P00736	*C1R*	[43,47]	High expression of complement components in omental adipose tissue
3	P00742	*F10*	[48,49]	Chronic low-grade inflammation, but is likely also due to direct effects of adipose tissue on mediators of coagulation
4	P01700	*IGLV1-47*	[50]	Differentially expressed gene in normal individuals and obese patients with breast cancer
5	P02655	*APOC2*	[51,52]	Cofactor for lipoprotein lipase, a plasma enzyme that hydrolyzes triglycerides/agent for obesity
6	P04278	*SHBG*	[46,53]	Decreased *SHBG* levels may be one of the components of the metabolic syndrome
7	P07358	*C8B*	[54]	Protein encoded by *C8B* gene and associated with complement activation was shared across diets indicating that a core set of proteins participate in tissue response to high-fat diet
8	P07360	*C8G*	[54]	Protein encoded by *C8G* gene and associated with complement activation was shared across diets indicating that a core set of proteins participate in tissue response to high-fat diet
9	P08185	*SERPINA6*	[55,56,57,58,59]	Corticosteroid-binding globulin polymorphism could influence obesity, metabolic, or hypothalamo-pituitary adrenal axis activity parameters
10	P0DJI8	*SAA1*	[60,61]	Major acute phase protein, correlating with obesity and insulin resistance in human
11	P10643	*C7*	[47]	Constituent of the membrane attack complex (MAC) that plays a key role in the innate and adaptive immune response by forming pores in the plasma membrane of target cells
12	P20742	*PZP*	-	*PZP* levels are individual-specific, do not correlate strongly with obesity
13	P22352	*GPX3*	[62,63,64,65,66]	*GPX3* expression is significantly higher in lean compared to obese as well as in insulin-sensitive compared insulin-resistant individuals with obesity
14	P25311	*AZGP1*	[39,40,41,67,68,69,70,71]	*AZGP1* stimulates lipid degradation in adipocytes and causes the extensive fat losses associated with some advanced cancers. May bind polyunsaturated fatty acids. Can promote the browning of white adipose tissue and can serve as a potential therapeutic target for treating metabolic diseases such as obesity. It is reduced in obesity, with a trend to further decrease with prediabetes and type 2 diabetes
15	P35858	*IGFALS*	[72]	*IGFALS* is involved in protein-protein interactions that result in protein complexes, receptor-ligand binding or cell adhesion. Children and adolescents with a variety of illnesses and metabolic disorders have altered circulating IGF-I and IGFBP levels. Circulating IGF and IGFBP levels overlap with normal values
16	P51884	*LUM*	[73]	*LUM* over-expression in visceral fat and liver resulted in improved insulin sensitivity and glucose clearance. Over-expression of *LUM* increases insulin sensitivity
17	Q06033	*ITIH3*	[43,74]	*ITIH3* negatively correlated with obesity
18	Q96KN2	*CNDP1*	[75]	An increased risk for obesity/overweight due to genotypes of *CNDP1* was observed only in the group with a low carotene/carbohydrate intake ratio. In the high carotene/carbohydrate intake group, the genotype of *CNDP1* was no risk factor for obesity/overweight

## Data Availability

The data presented in this study are available in PRIDE repository (dataset identifier PXD023526); Supplementary Materials are available via link https://zenodo.org/record/4432333#.YAbOw1X7QuU.

## Supplementary Materials

The following are available online at https://www.mdpi.com/2075-4426/11/2/64/s1, Table S1: Anthropometrical and clinical characteristic of individuals under study; File S1: Principal Components Analysis of clinical parameters and proteins identified in the samples of blood plasma.
