# Supplementary material for: Does Proteomic Mirror Reflect Clinical Characteristics of Obesity?
Source: J Pers Med. 2021 Jan 21;11(2):64. doi: 10.3390/jpm11020064 (PMC7912072; doi:10.3390/jpm11020064)
Supplement: Supplementary file 1 [file jpm-11-00064-s001.zip › Supplementary File S1.pdf]

Supplementary Note 1: Principal Components Analysis of clinical parameters and proteins identified in the samples of blood plasma

We performed a PCA to reduce dimensionality both for proteins and parameters of blood tests, which significantly differed between groups under study.

These analyses showed that there was no division into groups for both components, both for the parameters of the blood tests and for the mass spectrometrically identified proteins. A feature of the distribution along the PC1 axis can be distinguished, since patients accumulate in the range from -0.2 to 0.2, with the exception of the overweight (ow) group.

All patients are evenly spaced along the PC2 axis. The PCA in this case did not help to identify features of maximum importance.

For clinical parameters

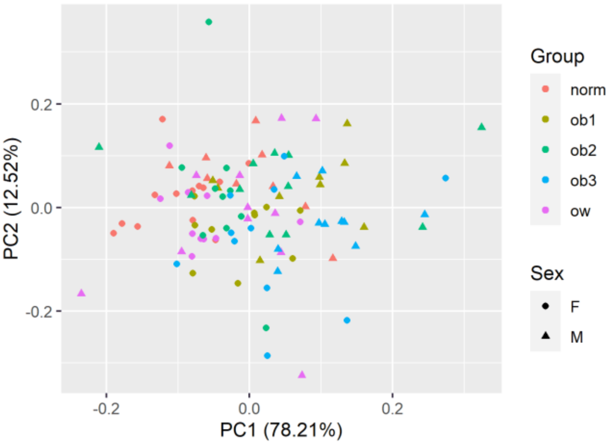

For proteins detected in the samples of blood plasma

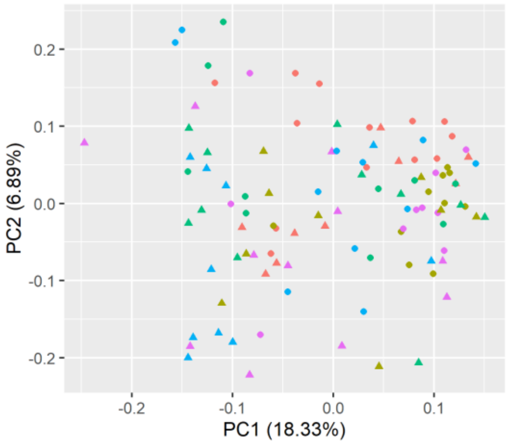

Legend: controls (NORM, BMI = 18.5-24.9), overweight patients (OW, BMI = 25.0-29.9), and patients with obesity stage 1 (OB1, BMI = 30.0-34.9), 2 (OB2, BMI = 35.0-39.9), and 3 (OB3, BMI > 40.0).
